# Study of the microstructure of chitosan aerogel beads prepared by supercritical CO_2_ drying and the effect of long-term storage

**DOI:** 10.1039/d2ra01875f

**Published:** 2022-07-21

**Authors:** Chun-gong Li, Qi Dang, Qinqin Yang, Dong Chen, Hongliang Zhu, Jiachen Chen, Runjin Liu, Xiang Wang

**Affiliations:** Key Laboratory of Biorheological Science and Technology, Ministry of Education, College of Bioengineering, Chongqing University Chongqing 400044 PR China xwangchn@vip.sina.com

## Abstract

In order to investigate the pore properties and effect of storage time on the microstructure of CO_2_-dried aerogels, chitosan aerogel beads were obtained from chitosan hydrogels with an initial concentration in the range of 1.5–3.0 wt% through SCCO_2_ drying and freeze-drying (as a comparison). The SCCO_2_-dried chitosan aerogels showed a three-dimensional network structure, and had higher BET surface area (200 m^2^ g^−1^) and higher crystallinity (0.62/XRD, 0.80/ATR-FTIR) than the freeze-dried aerogels. The stability of the microstructure of the SCCO_2_-dried chitosan aerogel beads during 10 months was studied. The BET surface area of the aerogel beads at each concentration declined by 30.5% at 2 months, 56.7% at 6 months and 67.2% at 10 months. Accelerated aging tests of the chitosan aerogel beads were carried out to study the effect of humidity on the chitosan aerogel beads. The average diameter of the chitosan aerogel decreased from 2.3 mm to 0.9 mm when stored at 65 °C with 90% relative humidity (RH). In contrast, there was no obvious change during storage at 65 °C with 20% RH. The amount of adsorbed water increased from 4% to 12% at 65 °C with 90% RH for 96 h, and the bound water content of the aerogel beads gradually increased. This study demonstrates that SCCO_2_-dried chitosan aerogel beads could be better at maintaining their mesoporous structure, and the adsorption of water from the surrounding air had a significant effect on the microstructure and shrinkage of the chitosan aerogel beads.

## Abstract 2

Chitosan aerogel beads prepared by different drying methods were compared, and the effects of long-term storage and humidity on the structure were investigated.

## Graphical abstract

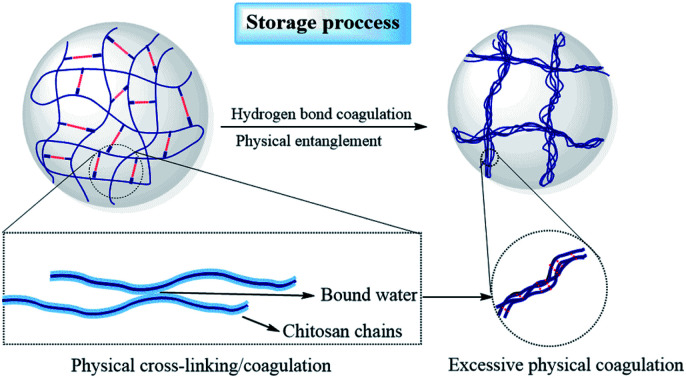

## Introduction

Aerogels are highly porous and sponge-like solid materials with high specific surface areas, ultralow thermal conductivities and extremely low densities.^1,2^ During the last decade(s), aerogels produced from biopolymers have attracted increasing interest not only due to the wide applications of porous functional materials but also due to the dream of renewable and sustainable feedstock.^3,4^ Chitosan, the second-most abundant and the only cationic biopolymer in nature,^3^ has become a hot topic in academic research due to its outstanding properties, such as biocompatibility,^5^ biodegradability,^6^ non-toxicity,^7^ antioxidant activity^8^ and antibacterial activity.^9^ Besides, chitosan has various chemical functional groups containing amino (–NH_2_) and hydroxyl (–OH) moieties, which is beneficial to the formation of aerogels *via* covalent crosslinks between –NH_2_ groups or hydrogen bonding and the entanglement of chains stimulated by pH changes or antisolvents.^10^ Therefore, there are a variety of potential applications for chitosan aerogels, including drug delivery systems,^11^ thermal and acoustic insulation,^12^ catalysis,^13^ sensing,^14^ CO_2_ capture,^15^ water purification,^16^ and hemoperfusion.^17^

The formation of chitosan aerogels usually goes through the following stages: chitosan dissolution, gelation and formation of the hydrogel, solvent exchange and solvent removal by a drying method.^3^ It has been confirmed that the preparation steps of the gel determine the formation of the porous framework, in which drying is the pivotal step to transform the wet gel into a porous solid. Supercritical drying is one of the widely used drying methods to produce aerogels, which involves crossing the critical point of the solvent by increasing the temperature and pressure, minimizing the damage to the microstructure (microporous structure) caused by the surface tension of the solvent.^12^ Supercritical carbon dioxide (SCCO_2_) is by far the most common solvent due to its appropriate conditions of critical pressure and temperature (31 °C, 7.38 MPa), which can prevent the degradation of the biopolymers.^4^ Freeze-drying is another common method to prepare aerogels, which involves crossing the liquid–gas phase boundary by freezing, with the ice crystals acting as templates of the pores,^3^ in which the size of ice crystals is directly related to the freezing speed, affecting the microstructure of the obtained aerogels. Ciftci *et al.* prepared lupin hull cellulose nanofiber aerogels by slow freeze-drying with a regular freezer and fast freeze-drying with liquid nitrogen or SCCO_2_ drying, and the results demonstrate that the SCCO_2_ drying method led to a 3D nanoporous network structure with a lower density and higher specific surface area and porosity compared with freeze-drying.^18^ Similar results have been found with other biological macromolecular aerogels, such as alginate^19^ and pectin.^20^ Therefore, it is necessary to investigate the microstructure of chitosan aerogels prepared by different drying methods.

After the formation of aerogels, they might have to undergo long-term storage in some actual applications; hence, it is critical for their further application to explore the changes in aerogel properties with storage time, which is easily overlooked in the application of biopolymer aerogels. It has been reported that the optical, barrier and mechanical properties have significant changes with the storage time of chitosan films, which involves the rearrangement of polymer chains during storage.^21^ The specific surface area under storage of alginate aerogels prepared with SCCO_2_ decreased obviously after 3 months.^19^ However, not much attention has been paid to the effect of storage time on the properties of chitosan aerogels, especially on their microstructure.

In this study, chitosan hydrogel beads were prepared with different initial concentrations of chitosan in the range of 1.5 wt% to 3 wt%, and were then dried by SCCO_2_ drying, fast freeze-drying and slow freeze-drying methods to obtain aerogel beads. In addition, the morphology, BET surface area and crystallization of the chitosan aerogel beads were evaluated by SEM, BET analysis, XRD and ATR-FTIR spectroscopy. Furthermore, the pore size distribution and BET surface area of SCCO_2_-dried chitosan aerogel beads were investigated after storage at room temperature in a sealed vessel for 10 months to evaluate the effect of storage time on the microstructure of SCCO_2_-dried aerogels, which provided adequate proof for further applications.

## Experimental section

### Materials

Chitosan (viscosity: 100–200 mPa s^−1^) with a deacetylation degree above 95.0% was purchased from Aladdin Reagent Co., Ltd. (Shanghai, China). Ethyl alcohol (AR), acetate acid (AR), sodium hydroxide (NaOH, AR) and normal saline were supplied by Chuandong Co., Ltd. (Chongqing, China).

### Preparation of chitosan hydrogel beads

**Fig. 1:**
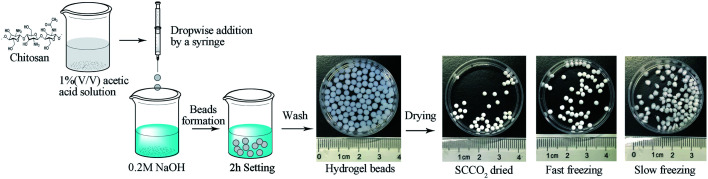
Steps of producing the chitosan aerogel beads.

The synthetic procedures of the chitosan aerogel beads are shown in Fig. 1. The chitosan reagent was proportionately dissolved in a 1% (v/v) acetic acid solution to obtain 1.5 wt%, 2.0 wt%, 2.5 wt%, and 3 wt% chitosan solutions at room temperature, and the bubbles in the sol were removed by vacuum degassing. The obtained chitosan sol was then dipped into a 0.2 M NaOH solution using a constant current pump with stirring to produce chitosan hydrogel beads. After setting for 2 h, the chitosan hydrogel beads were washed with deionized water until neutral pH for the next step of drying to produce the aerogel beads.

### Preparation of chitosan aerogel beads by SCCO_2_ drying

The chitosan hydrogel beads were soaked in 30%, 50%, 70%, and 100% (v/v) ethanol for 1 h and set in 100% ethanol for 24 h to make sure that the water in the chitosan hydrogel beads was completely replaced with ethanol. Then, SCCO_2_ drying was used to remove the ethanol from the alcogels, which was carried out using a Leica EM CPD300 Critical Point Dryer (Leica, Germany). Specifically, the chitosan alcogel beads were placed into the pressure vessel and sealed after being added to an excess amount of ethanol (40 mL). Then, the temperature of the pressure vessel was cooled from room temperature (25 °C) to the set temperature (15 °C), and the pressure was increased to 50 bar by flowing CO_2_ into the pressure vessel. After that, ethanol was extracted *via* CO_2_ circulation for 15 cycles at 50 bar for 1 h. Finally, the temperature was increased to 35 °C and the pressure vessel was depressurized to atmospheric pressure to obtain the SCCO_2_-dried aerogel beads. The chitosan aerogel beads were collected from the pressure vessel and stored in a hermetic glass vessel at room temperature for further characterization.

### Preparation of aerogel beads by freeze-drying

Before the freeze-drying process, chitosan hydrogel beads were placed in a vessel and frozen by fast freezing with an excess amount of liquid nitrogen in a short time or by slow freezing with a regular freezer at −18 °C for 24 h. After that, the frozen chitosan hydrogel beads were transferred to a freeze dryer and sublimated at −60 °C and 15 Pa for 48 h to obtain fast freeze-dried aerogel beads and slow freeze-dried aerogel beads. Finally, the chitosan aerogel beads were kept in a hermetic glass vessel at room temperature for further analysis.

### Storage of the SCCO_2_-dried aerogel beads

A known amount of SCCO_2_-dried aerogel beads at different concentrations were placed in hermetic glass vessels. All samples were stored in the dark at room temperature (average 24 °C and 60% relative humidity) and were sampled for analysis after storage for 2 months, 6 months and 10 months.

### Accelerated aging test

Accelerated aging tests of the chitosan aerogel beads were carried out to study the effect of humidity on the aerogel beads. 3 wt% aerogel beads were stored at 65 °C and 90% relative humidity or 65 °C and 20% relative humidity. Then, the average diameters of aerogel beads stored for 0.5 h, 1 h, 2 h, 3 h, 12 h, 24 h and 96 h were measured, and the corresponding aerogel beads were sampled for thermogravimetric (TG) analysis.

### Characterization

The morphologies of the chitosan aerogel beads were observed by scanning electron microscopy (SEM, Zeiss EVO18) after coating with gold. The specific surface area, pore volume and pore size of the chitosan aerogel beads were characterized by Brunauer–Emmett–Teller (BET) measurement and Barrett–Joyner–Halenda (BJH) measurement using a surface area analyzer (Quadrasorb 2 MP, Quantachrome Instruments, USA). The crystallinity of the chitosan aerogel beads was determined using either an XRD diffractometer (Empyrean, Spectris Pte. Ltd, Netherlands) or a Fourier-transform attenuated total reflection infrared (ATR-FTIR) spectrometer (Nicolet iN10, Thermo, US). The XRD patterns were measured in the region of 2*θ* = 5°–45° at a scanning speed of 10° min^−1^ with Cu Kα radiation at 40 kV and 40 mA. The crystallinity indices (CrI) of the chitosan aerogel beads and the chitosan reagent were determined by XRD using the following equation:1
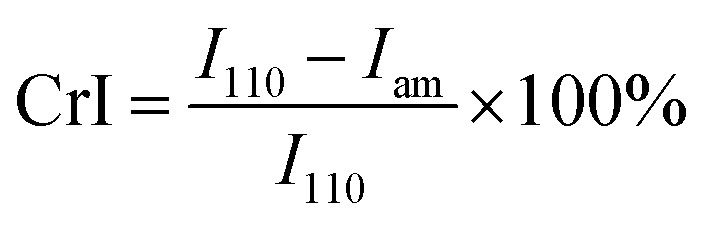
where *I*_110_ and *I*_am_ are the maximum intensity (2*θ* = 20°) of the (110) lattice diffraction and the intensity of amorphous diffraction at 2*θ* = 16° of chitosan, respectively.^22^ The ATR-FTIR spectra were recorded between 4000 and 700 cm^−1^ with 128 scans at a normal resolution of 4 cm^−1^. The CrI of the chitosan aerogel beads and the chitosan reagent were determined by ATR-FTIR using the ratio of absorbances at *A*_1382_ cm^−1^/*A*_2920_ cm^−1^.^23^ Thermogravimetric (TG) analyses were performed on a Mettler TGA 2 TG analyzer (Mettler Toledo, Switzerland) with a heating rate of 10 °C min^−1^ from 30 °C to 800 °C under N_2_.

## Results and discussion

### Morphology

#### SCCO_2_-dried chitosan aerogel beads

**Fig. 2:**
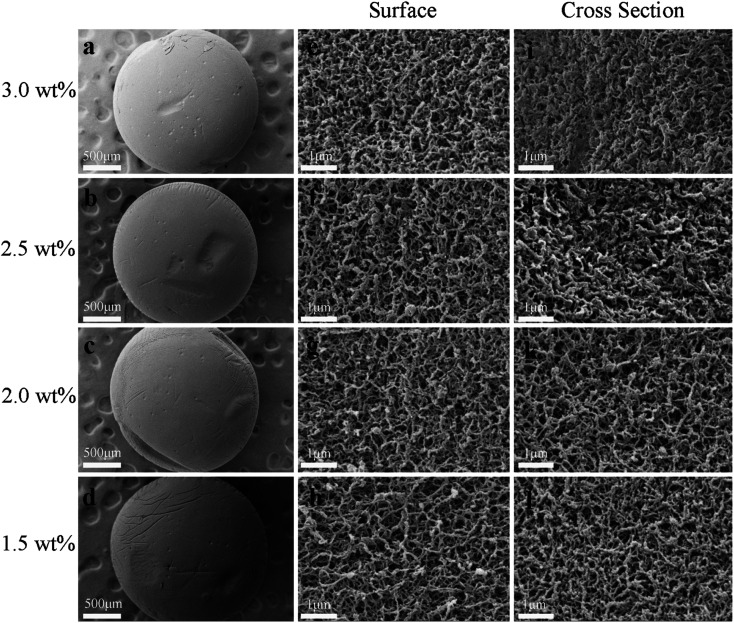
Morphologies of the SCCO_2_-dried chitosan aerogels at different concentrations: appearance (a–d), surface (e–h) and cross-section (i–l).

The morphologies of the SCCO_2_-dried chitosan aerogel beads with different initial chitosan concentrations are shown in Fig. 2. It was observed that the chitosan aerogel beads retained the shape of a sphere with a diameter of about 1.8 mm (Fig. 2a–d), with no obvious difference in the chitosan beads of different initial concentrations, indicating that the SCCO_2_-dried chitosan aerogel beads had enough strength to maintain their spherical shape at an initial chitosan concentration ranging from 1.5 wt% to 3 wt%. High-magnification images of the surface of the aerogel beads show the three-dimensional network with a highly dense, homogeneous and porous structure observed either on the surface (Fig. 2e–h) or in cross section (Fig. 2i–l) at each concentration level. However, the pore structure of the aerogels became denser, and the chain of the chitosan polymer was aggregated more densely, as the initial concentration increased from 1.5 wt% to 3 wt%, especially in the cross-section, which might be attributed to the increased attraction between chitosan molecular chains due to the formation of more hydrogen bonds or even physical entanglement between the functional groups on the chitosan chain.^4^

#### Freeze-dried chitosan aerogel beads

**Fig. 3:**
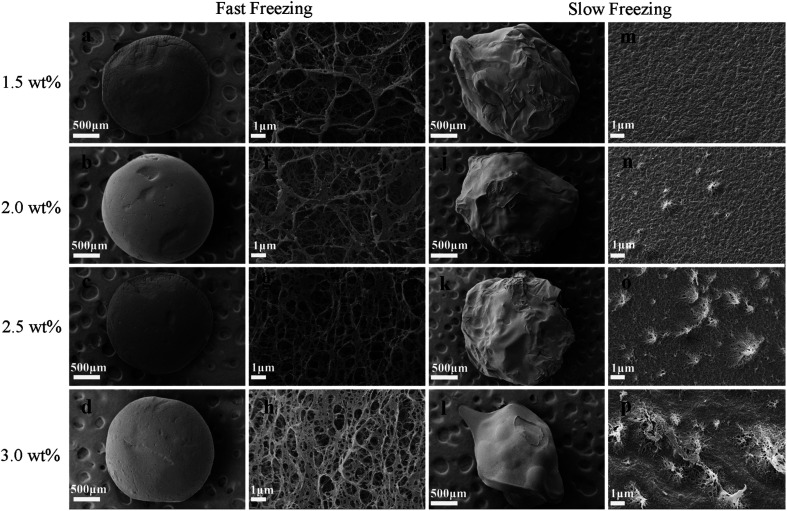
Morphologies of the fast-dried chitosan aerogels at different concentrations: appearance (a–d) and surface (e–h), and the morphologies of the slow-dried chitosan aerogels at different concentrations: appearance (i–l) and surface (m–p).

The morphologies of the fast freeze-dried and slow freeze-dried aerogel beads with different initial chitosan concentrations are shown in Fig. 3. The fast freeze-dried aerogel beads maintained a regular spherical shape and the surface was smooth (Fig. 3a–d), while the slow freeze-dried aerogel beads had obvious shrinkage, exhibiting irregular spheres (Fig. 3i–l). Furthermore, it was observed from the high-magnification images that the fast freeze-dried aerogel beads presented a random and porous network structure with a few sheet-like structures on the outer surface (Fig. 3e–h), but the pores of the slow freeze-dried aerogel beads almost completely disappeared with whole and compact sheet-like structures (Fig. 3m–p), indicating that the structure of the slow freeze-dried aerogels was severely collapsed. These results demonstrate that fast freeze-drying with liquid nitrogen could maintain the structure of the aerogel beads better than slow freeze-drying, which might be attributed to the scale and distribution of the ice crystals formed during freezing. In general, there were two major stages during the freeze-drying method. The first stage was the formation of ice crystals between the chitosan polymers *via* freezing, and the next stage was the sublimation of ice.^23^ The freezing speed at the first stage is important for the formation of ice crystals, affecting the scale and distribution of the ice crystals in hydrogels.^24^ The ice crystals grow little by little during the slow freeze-drying process, resulting in the continuous destruction of the skeletal structure of the hydrogel, and the aerogels will collapse after the sublimation of ice, leading to shrinkage, as shown in Fig. 3i–l. In comparison, microcrystals are formed quickly under fast freeze-drying by adding liquid nitrogen at a temperature of −196 °C, resulting in smaller ice crystals that decrease the damage of crystals to the skeletal structure of the hydrogel. Besides, smaller pore structures and more compact two-dimensional sheet-like structures were observed as the concentration increased from 1.5 wt% to 3.0 wt%, which was the result of the increased hydrogen bonding interaction of chitosan fibers.

In summary, the drying methods had significant effects on the pore structure of the chitosan aerogels. Compared with the slow freeze-dried aerogel beads, the SCCO_2_-dried and fast freeze-dried aerogel beads exhibited porous structures with a spherical shape. Although porous structures were displayed in both the SCCO_2_-dried and fast freeze-dried chitosan aerogel beads, the fast freeze-dried aerogels presented more macroporous structures than the SCCO_2_-dried aerogels. Besides, the SCCO_2_-dried aerogels showed three-dimensional pore structures, while the fast freeze-dried aerogels presented mostly two-dimensional sheet-like pore structures. However, these analyses are only based on a subjective visual evaluation of the SEM images; hence, it is necessary to further study the properties of the chitosan aerogels prepared by SCCO_2_ drying and fast freeze-drying.

### Characterization of the chitosan aerogels

**Fig. 4:**
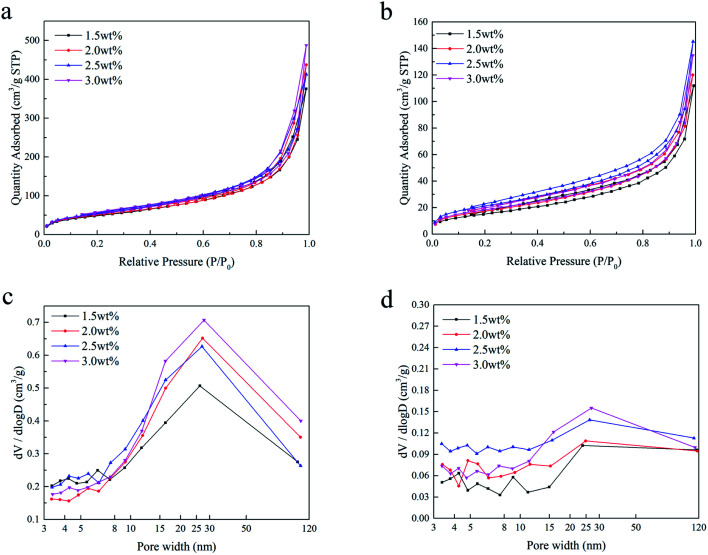
N_2_ adsorption/desorption isotherms of the SCCO_2_-dried aerogels (a) and fast freeze-dried aerogels (b), and the pore size distribution of the SCCO_2_-dried aerogels (c) and the fast freeze-dried aerogels (d) at different concentrations.

Fig. 4 shows the nitrogen adsorption/desorption isotherms and the corresponding pore size distributions of the SCCO_2_-dried aerogels and fast freeze-dried aerogels. As shown in Fig. 4a and b, a hysteresis loop occurred on the desorption isotherm, indicating that the mesopores and macropores within the aerogel beads prepared by the SCCO_2_ drying and fast freeze-drying methods were dominant at a relatively high pressure. In addition, the BET surface area calculated by the adsorption/desorption isotherms ([0.08–0.3]*p*/*p*_0_, *σ*N_2_ = 0.162 nm^2^) and the pore volume analyzed by the Barrett–Joyner–Halenda (BJH) method are shown in Table 1. The BET surface area and BJH pore volume of the SCCO_2_-dried aerogel beads were higher than those of the freeze-dried aerogel beads at each concentration level, indicating that SCCO_2_ drying could maintain the pore structure better during the formation of aerogels, which could avoid the collapse within the small pores caused by liquid–gas surface tension and liquid–solid adhesive forces.^4^ The BET surface area of the slow-dried aerogels was too low and was close to the detection limit of the N_2_ adsorption–desorption equipment; thus, it was not significant to compare the difference between the different concentrations, indirectly reflecting the poor pores maintained in the slow-dried aerogels. In addition, it is interesting that the aerogel beads prepared by the SCCO_2_ drying and freeze-drying methods both had the largest BET surface area at a concentration of 2.5 wt%, which might be attributed to the hydrogen bonding interactions reaching their threshold, leading to a decline in the BET surface area.^25^ A similar trend had been reported in other studies.^26^

**Table 1:** Comparison of the properties of the chitosan aerogel beads obtained *via* different drying methods

Property	SCCO_2_-dried				Fast freeze-dried				Slow freeze-dried			
	1.5 wt%	2 wt%	2.5 wt%	3 wt%	1.5 wt%	2 wt%	2.5 wt%	3 wt%	1.5 wt%	2 wt%	2.5 wt%	3 wt%
BET surface area (m^2^ g^−1^)	179	183	200	195	56	63	78	69	2.9	1.4	0.5	1.6
BJH pore volume (cm^3^ g^−1^)	0.544	0.64	0.603	0.715	0.108	0.129	0.204	0.178	0.021	0.008	0.012	0.003

Furthermore, it can be found from Fig. 4c and d that the pore size distributions of the SCCO_2_-dried aerogels and fast freeze-dried aerogels are not limited to mesopores (2–50 nm) and clearly trespass into the domain of macroporosity (>50 nm), in which the pore size distribution of the SCCO_2_-dried chitosan aerogels was more concentrated in 20–40 nm, while the fast freeze-dried aerogels were more dispersed. This result is consistent with the results observed by SEM and demonstrates that although freeze-drying could maintain the porosity of gels, ice crystals destroyed the skeletal structure of the gel, generally resulting in more macroporous sheet-like structures, and the CO_2_ supercritical fluid replaced ice crystals that acted as templates of the pores, thereby avoiding the occurrence of the above phenomenon, ultimately obtaining more mesoporous structures.

### Crystallinity

The crystallinity is related to the mechanical properties, thermal stability and permeability of materials. In order to evaluate the impact of the two drying methods on the crystal structures of the aerogel beads, the XRD patterns and ATR-FTIR spectra were recorded.

**Fig. 5:**
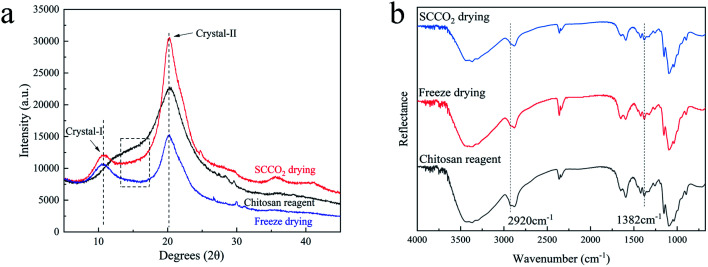
XRD patterns (a) and ATR-FTIR spectra (b) of the chitosan reagent and chitosan aerogels prepared by SCCO_2_ drying and fast freeze-drying.

In generally, the XRD patterns included the crystalline regions and amorphous regions of chitosan.^27^Fig. 5a shows a comparison between the X-ray diffraction patterns of the chitosan reagent and aerogel beads. For both the aerogel beads dried by SCCO_2_ drying or fast freeze-drying, a new peak at 2*θ* = 10°–11° appeared compared with the chitosan reagent, which could be contributed by the crystal-I of the crystalline regions of the chitosan molecules.^28^ Meanwhile, the characteristic peaks of the chitosan reagent at 2*θ* = 20°–21° were observed, and the peaks at 2*θ* = 20°–21° of the chitosan reagent became much more broad than those of the aerogel beads, which could be attributed to the amorphous structure of the chitosan reagent,^29^ indicating that the original chitosan chain has been reorganized during the formation of the aerogels.^30^ As is well known, the crystallinity index (CrI) reflects the regular pattern of the aggregation state for polymer chains and the CrI of the chitosan aerogel beads and reagent were calculated following the strategy of Focher *et al.*^31^ It was noted that the CrI followed the order: SCCO_2_ drying (CrI = 0.62) > freeze-drying (CrI = 0.47) > chitosan reagent (CrI = 0.38). Furthermore, compared to the chitosan reagent, the XRD spectra of the SCCO_2_-dried and fast freeze-dried aerogel beads in the 20°–30° range were smoother, which might be related to the higher regularity of the aggregation of the polymer chains in the SCCO_2_-dried and fast freeze-dried aerogel beads. These results indicate that: (i) SCCO_2_ drying and freeze-drying significantly affect the XRD patterns and relative crystallinity of chitosan; (ii) the SCCO_2_-dried aerogels might be more homogeneous than the freeze-dried aerogels due to their higher CrI. Similar results have been obtained for other polysaccharides.^32^

The ATR-FTIR spectra of the chitosan aerogels were compared with those of the chitosan reagent, as shown in Fig. 5b. Although the positions of the peaks in the ATR-FTIR spectra of all the samples were the same, the adsorption strength was slightly different, for which the ratio of absorbance at *A*_1382_ cm^−1^/*A*_2920_ cm^−1^ could be used to evaluate the CrI of the aerogel samples and the chitosan reagent.^22,31^ In addition, the CrI followed the order: SCCO_2_ drying (CrI = 0.80) > freeze-drying (CrI = 0.77) > chitosan reagent (CrI = 0.69). The variation trend of CrI was consistent with that of the XRD determinations. Furthermore, all peaks in the ATR-FTIR spectra of the SCCO_2_-dried aerogels and fast-dried aerogels became more sharp than those of the chitosan reagent, probably associated with the more consistent hydrogen bonding environments between the functional groups of –NH_2_, –OH, and C=O in the aerogel samples,^10^ especially obvious in the ATR-FTIR spectra of the SCCO_2_-dried aerogels.

### Effect of storage on the morphology and microstructure of the SCCO_2_-dried aerogels

**Fig. 6:**
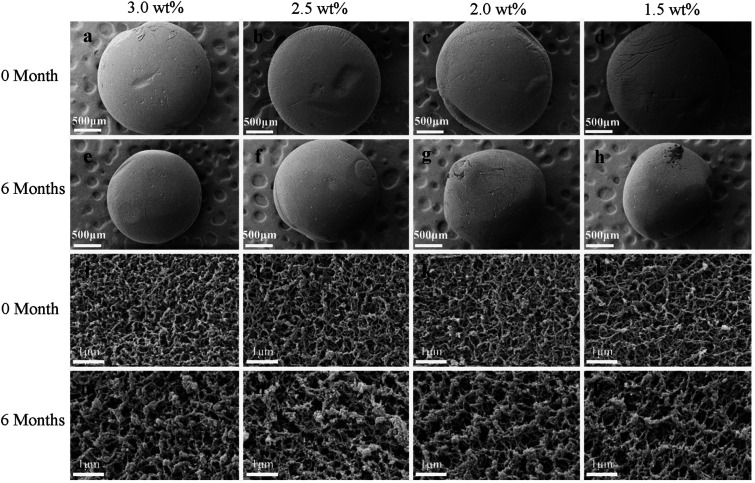
Morphologies of the SCCO_2_-dried aerogel beads after storage for 0 and 6 months: appearance (0 month, (a–d) and 6 months, (e–h)) and surface (0 month, (i–l) and 6 months, (m–p)).

Fig. 6 shows the SEM images of the SCCO_2_-dried aerogels after storage for 0 and 6 months. It can be observed from Fig. 6a–h that the diameters of the chitosan aerogel beads at each initial concentration level decreased after storage for 6 months, implying that the internal structure collapsed during storage. Furthermore, it can be found from the high-magnification images (Fig. 6i–p) that the nanofiber-like chitosan chains of the aerogels showed obvious aggregation after storage for 6 months, resulting in the thin and uniform chitosan fiber bundles becoming thicker over time, which might be attributed to the continuous collapse of chitosan fibers caused by hydrogen bonding interactions between the nanofibers during storage. This suggests that physical coagulation can not only occur in the stage of CO_2_ supercritical drying to form nanofiber-like structures but can continue to occur slowly during subsequent storage.

**Fig. 7:**
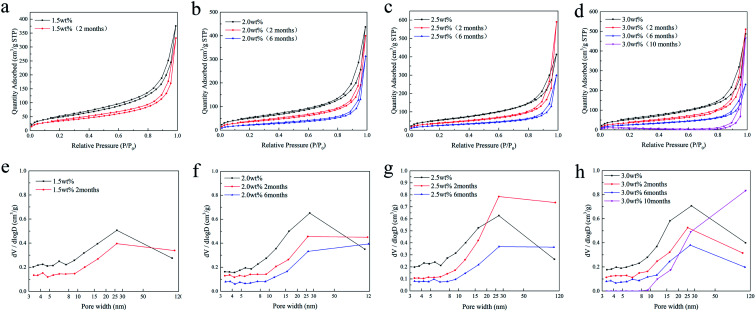
N_2_ adsorption/desorption isotherms (a–d) and the pore size distributions (e–h) of the SCCO_2_-dried chitosan aerogels at different concentrations.

Fig. 7 shows the adsorption/desorption isotherms and the corresponding pore size distribution of the SCCO_2_-dried chitosan aerogel beads at different concentrations ranging from 1.5 to 3.0 wt% after storage. It can be observed from Fig. 7a–d that all samples present a curve along with a hysteresis loop related to the existence of mesopores. Under moderate relative pressure (*P*/*P*_0_), the adsorption capacity of all aerogel beads gradually decreases as the storage time increases, which indicates that mesopores are decreasing.

Furthermore, Fig. 7e–h show that the BJH pore volume of the chitosan aerogel beads decreased with storage time, suggesting the shrinkage and skeletal collapse of the aerogels. Specifically, the BJH pore volume of the smaller mesopores (<25 nm) of the aerogel beads at each concentration declined faster than the larger mesopores (>25 nm), in which the smaller mesopores (<10 nm) of 3 wt% beads disappeared after storage for 10 months.

**Fig. 8:**
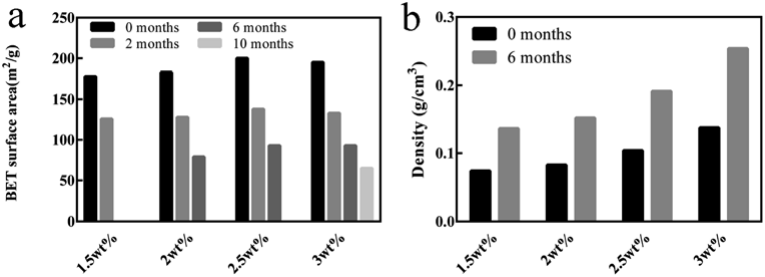
BET surface area (a) and density (b) of the chitosan aerogel beads (SCCO_2_-dried) at different concentrations after storage.

Besides, the BET surface area and density of the chitosan aerogel beads at each concentration during storage were calculated, and are shown in Fig. 8. It is obvious that the BET surface area of all samples decreased over time (Fig. 8a), in which the BET surface area of the chitosan aerogel beads presented a declining rate of 30.5% at 2 months, 56.7% at 6 months and 67.2% at 10 months, which might be due to the disappearance of mesopores and the increment of macropores caused by physical coagulation, and similar results have been shown for other polysaccharides.^19^ Although the BET surface area of the SCCO_2_-dried aerogels decreased after storage for 6 months, it was still higher than that of the freeze-dried aerogels. Meanwhile, as shown in Fig. 8b, the density of the chitosan aerogel beads at each concentration increased after 6 months of storage due to the decrease in diameter over time.

**Fig. 9:**
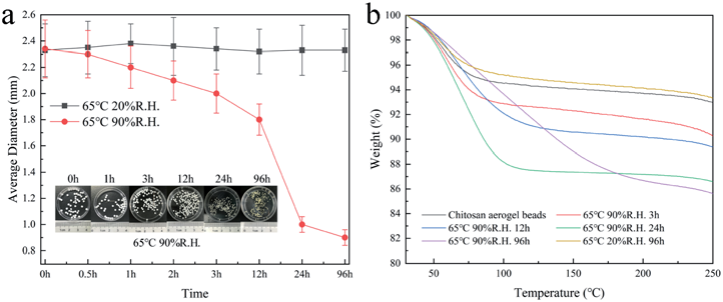
Average diameter (a) and TGA curves (b) of the chitosan aerogel beads.

**Fig. 10:**
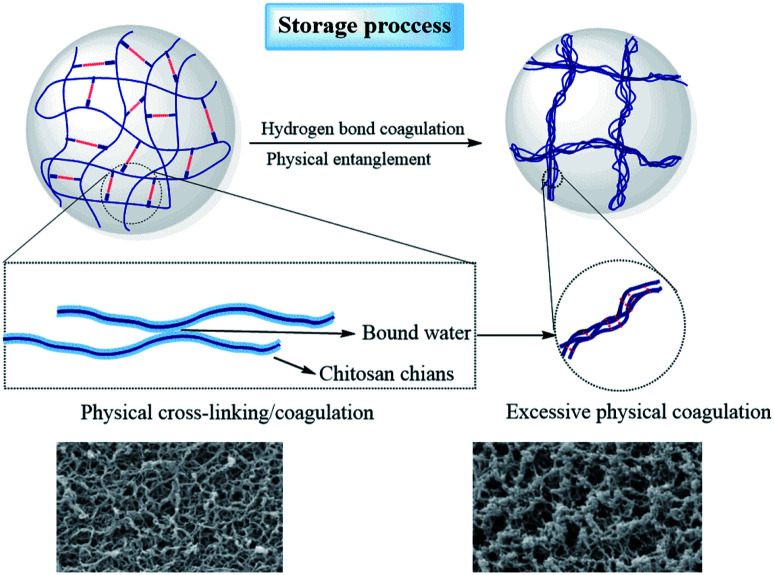
Proposed mechanisms for the changes in the microstructure of the SCCO_2_-dried chitosan aerogels during storage.

To further evaluate the effect of the adsorption of water from the surrounding air on the properties of the aerogel beads, accelerated aging tests of the chitosan aerogel beads were performed at a relatively high temperature and high humidity. As shown in Fig. 9a, the average diameter of the chitosan aerogel beads decreased rapidly within 24 h and then gradually tended to equilibrium when stored at 65 °C with a relative humidity of 90%. In contrast, there were no obviously changes in the average diameter of the aerogel beads during storage at 65 °C with a relative humidity of 20%. These results indicate that the high relative humidity was more likely to cause the shrinkage of the chitosan aerogel beads and was not conducive to the preservation of the chitosan aerogels, which might be attributed to the humidity uptake of aerogels during storage caused by the hydrophilic backbone and high specific area of the chitosan aerogel beads. TG analyses were performed to evaluate the amount of humidity uptake. As shown in Fig. 9b, the loss ratio of the untreated aerogel beads in weight was about 5%, and increased to about 12% after storage at 65 °C with 90% RH for 96 h. In contrast, the weight loss rate of the aerogel beads when stored at 65 °C with 20% RH for 96 h decreased to about 4%, which was probably due to the relatively low humidity. In addition, the weight loss at 30 °C to 200 °C was mostly due to moisture removal.^26^ These results prove the humidity uptake during storage at high relative humidity. Moreover, the water weight loss of the untreated aerogel beads occurred mainly in the range of 30 °C to 100 °C, but the water weight loss of the aerogel beads stored at 65 °C with 90% RH was in the range of 30 °C to 200 °C, which implied that the bound water content of the aerogel beads gradually increased. Higher humidity caused the increment of bound water on the surface of the chitosan fibers, which resulted in an increase in the interaction force between the chitosan fibers, finally leading to shrinkage of the chitosan aerogel beads.^33^ These results suggest that hydrogen bonding cross-linking between the chitosan fibers mediated by bound water causes excessive physical coagulation of the chains in the chitosan aerogels, resulting in the disappearance of smaller mesopores (<10 nm), and ultimately leading to a decrease in the BET surface area, which is shown in Fig. 10. Therefore, when chitosan aerogels are stored for a long time, the removal of bound water on the surface of the chitosan fibers and maintaining a strict dry environment are necessary.

## Conclusions

In this work, chitosan aerogel beads were successfully fabricated by a SCCO_2_ drying method with different initial chitosan hydrogel concentrations and compared with freeze-dried chitosan aerogels, in which the SCCO_2_-dried aerogels presented a three-dimensional nanofiber-like porous network structure dominated by mesopores and had a higher BET surface area. The porous structure of the SCCO_2_-dried aerogels and fast freeze-dried aerogels became denser as the initial concentration increased, with the highest BET surface area at a concentration of 2.5 wt%. For the freeze-drying method, different cooling rates had a significant influence on the structure of the chitosan aerogel beads, in which the fast-dried aerogels could maintain their porous structure better than the slow-dried aerogels. Besides, the crystallinity of the SCCO_2_-dried aerogels was higher than that of the fast-dried aerogels, suggesting that the SCCO_2_-dried aerogels might possess good mechanical, stability and permeability properties. Last but not least, humidity during storage had significant effects on the microstructure of the chitosan aerogels. These results demonstrate that the SCCO_2_-dried chitosan aerogel beads could better maintain their mesoporous structure, and the effect of long-term storage on the microstructure could not be ignored.

## Author contributions

Chun-gong Li: Conceptualization, Methodology, Investigation, Data curation, Writing – original draft. Qi Dang: Methodology, Investigation, Writing – review & editing, Supervision, Formal Analysis. Qingqing Yang: Methodology, Investigation, Writing – review & editing, Supervision, Formal Analysis. Dong Chen: Methodology, Investigation, Writing – review. Hongliang Zhu: Writing – review. Jiacheng Chen: Writing – review. Runjin Liu: Writing – review. Xiang Wang: Supervision, Project administration, Visualization, Funding acquisition.

## Conflicts of interest

There are no conflicts to declare.

## Supplementary Material
